# A proteome-wide association study identifies putative causal proteins for breast cancer risk

**DOI:** 10.1038/s41416-024-02879-1

**Published:** 2024-10-28

**Authors:** Tianying Zhao, Shuai Xu, Jie Ping, Guochong Jia, Yongchao Dou, Jill E. Henry, Bing Zhang, Xingyi Guo, Michele L. Cote, Qiuyin Cai, Xiao-Ou Shu, Wei Zheng, Jirong Long

**Affiliations:** 1 Division of Epidemiology, Department of Medicine, Vanderbilt Epidemiology Center, Vanderbilt-Ingram Cancer Center, Vanderbilt University Medical Center, Nashville, TN USA; 2 Lester and Sue Smith Breast Center, Baylor College of Medicine, Houston, TX 77030 USA; 3 Department of Molecular and Human Genetics, Baylor College of Medicine, Houston, TX 77030 USA; 4 Indiana University Simon Comprehensive Cancer Center, Indianapolis, IN USA

**Keywords:** Cancer epidemiology, Breast cancer

## Abstract

**Background:**

Genome-wide association studies (GWAS) have identified more than 200 breast cancer risk-associated genetic loci, yet the causal genes and biological mechanisms for most loci remain elusive. Proteins, as final gene products, are pivotal in cellular function. In this study, we conducted a proteome-wide association study (PWAS) to identify proteins in breast tissue related to breast cancer risk.

**Methods:**

We profiled the proteome in fresh frozen breast tissue samples from 120 cancer-free European-ancestry women from the Susan G. Komen Tissue Bank (KTB). Protein expression levels were log2-transformed then normalized via quantile and inverse-rank transformations. GWAS data were also generated for these 120 samples. These data were used to build statistical models to predict protein expression levels via cis-genetic variants using the *elastic net* method. The prediction models were then applied to the GWAS summary statistics data of 133,384 breast cancer cases and 113,789 controls to assess the associations of genetically predicted protein expression levels with breast cancer risk overall and its subtypes using the S-PrediXcan method.

**Results:**

A total of 6388 proteins were detected in the normal breast tissue samples from 120 women with a high detection false discovery rate (FDR) *p* value < 0.01. Among the 5820 proteins detected in more than 80% of participants, prediction models were successfully built for 2060 proteins with R > 0.1 and *P* < 0.05. Among these 2060 proteins, five proteins were significantly associated with overall breast cancer risk at an FDR *p* value < 0.1. Among these five proteins, the corresponding genes for proteins COPG1, DCTN3, and DDX6 were located at least 1 Megabase away from the GWAS-identified breast cancer risk variants. COPG1 was associated with an increased risk of breast cancer with a *p* value of 8.54 × 10^–4^. Both DCTN3 and DDX6 were associated with a decreased risk of breast cancer with *p* values of 1.01 × 10^–3^ and 3.25 × 10^–4^, respectively. The corresponding genes for the remaining two proteins, LSP1 and DNAJA3, were located in previously GWAS-identified breast cancer risk loci. After adjusting for GWAS-identified risk variants, the association for DNAJA3 was still significant (*p* value of 9.15 × 10^–5^ and adjusted *p* value of 1.94 × 10^–4^). However, the significance for LSP1 became weaker with a *p* value of 0.62. Stratification analyses by breast cancer subtypes identified three proteins, SMARCC1, LSP1, and NCKAP1L, associated with luminal A, luminal B, and ER-positive breast cancer. NCKAP1L was located at least 1Mb away from the GWAS-identified breast cancer risk variants. After adjusting for GWAS-identified breast cancer risk variants, the association for protein LSP1 was still significant (adjusted *p* value of 6.43 × 10^–3^ for luminal B subtype).

**Conclusion:**

We conducted the first breast-tissue-based PWAS and identified seven proteins associated with breast cancer, including five proteins not previously implicated. These findings help improve our understanding of the underlying genetic mechanism of breast cancer development.

## Introduction

Breast cancer was the most common cancer diagnosed, and the second cause of cancer-related deaths among females in the United States in 2023 [1]. Globally, breast cancer remains the most common cancer and the leading cause of cancer-related deaths among women [2]. Genetic factors play an important role in the etiology of both familial and sporadic breast cancer [3]. To date, common genetic variants associated with breast cancer risk have been identified in approximately 200 common genetic loci through genome-wide association studies (GWAS) [3–12]. Large numbers of potential risk genes associated with breast cancer have been identified employing expression quantitative trait loci (eQTL) and transcriptome-wide association studies (TWAS) [3, 13–18]. However, these previous studies did not consider post-transcriptional and translational modifications.

Proteins play a vital role in orchestrating various important activities, such as metabolism and molecule transportation. Evidence from observational studies shows that circulating levels of some proteins, inclusive of C-reactive proteins, were associated with the risk of breast cancer [19]. Studies investigating protein quantitative trait loci (pQTL) have elucidated that single nucleotide polymorphisms in cis-regions (cis-SNPs) regulate protein levels in various tissues, including the brain, lung, blood, and liver [20–23]. Adapting the concept of TWAS [24], the proteome-wide association study (PWAS) framework, which incorporates pQTLs into GWAS data of the phenotype of interest [4], was developed [21]. PWAS aids in understanding the genetic architecture of the proteome and its overlap with gene expression and complex traits [21]. Through the application of this methodology, plasma protein biomarkers, particularly for breast cancer, were discovered [25–27]. However, the wide-ranging origins of blood proteins from multiple tissues may compromise the sensitivity and specificity of the detection of breast cancer biomarkers. Expression levels of many proteins are tissue specific [21]. Identifying protein expression levels in breast tissue can substantially enhance our understanding of the mechanisms underpinning breast cancer development and fortify drug development for breast cancer.

We describe here the first breast-tissue-based PWAS for breast cancer risk. In this study, we used proteomic data generated in normal female breast tissues and germline genomic data to build protein prediction models [28] and evaluated the associations of genetically predicted breast tissue protein levels with the risk of breast cancer overall and by subtypes among individuals of European ancestry utilizing S-PrediXcan [29]. The findings could inform future drug development to reduce disease burden and improve understanding of breast cancer carcinogenesis.

## Materials and methods

### Dataset

Normal breast tissues of cancer-free donors were obtained from the Susan G. Komen Tissue Bank (KTB). Details of the KTB have been described elsewhere [28]. The KTB was established to acquire breast tissue samples from volunteer donors not diagnosed with any cancer. The sample collection mostly happens at the Indiana University IU Simon Cancer Center in Indianapolis, IN, where five tissue collection events are held annually. During each event, about one hundred women donate their tissue samples. The sample collections are bounded by the approval of the Indiana University Institutional Review Board. KTB has collected breast tissue samples from 4932 cancer-free women as of 08/03/2024 (latest number of donors can be found: https://komentissuebank.iu.edu). In this study, we analyzed 120 cancer-free breast tissue samples from women of European ancestry (EA). Their ancestry was self-reported and genetically determined, with the European proportion ranging from 96.29 to 100.00%, using the software Admixture [30] with 1000 Genome [31] data as the reference. We also performed principal component analysis (PCA) using PLINK [32] and our data clustered with European ancestry samples from 1000 Genome [31].

### Breast tissue proteomics data profiling—Tandem mass tags quantitation

Frozen breast tissue samples were homogenized in a urea, SDS, and TEAB-based buffer, sonicated, and then cleaned up. Protein concentration was determined using a BCA assay. Each sample (100 μg) underwent STrap MS preparation, including reduction, alkylation, quenching, and overnight Trypsin/Lys-C digestion. Digests were eluted, dried, and reconstituted in HEPES pH8.5. A pooled composite sample was created for Tandem Mass Tags (TMT) bridging. TMT labeling was applied to all samples, followed by a one-hour incubation. The samples and a TMT-labeled composite were checked via nano LC-MS/MS. After further combining with the composite, samples were fractionated by HPLC. 12 fractions from each group were reconstituted and randomized for LC-MS/MS analysis. TMT quantification was performed with Proteome Discoverer 2.5 within-group normalization. In shotgun proteomics, peptides often map to multiple proteins due to sequence similarities. The parsimonious approach is widely used for protein quantification, selecting the protein with the most evidence while ignoring isoforms without unique peptides [33]. This study applied this method, with each protein group represented by a single quantified protein.

### Genotype data quality control and processing

Genotype data for this study was generated using Illumina Multi-Ethnic Genotyping Array (MEGA) by VANTAGE at Vanderbilt University Medical Center. Quality control (QC) procedures were performed in the same manner as the previous study [5]. Before imputation, we excluded SNPs with call rate <95%, consistency rate<95% among QC samples, inconsistent allele frequency with the 1000 Genome European data [31] (> 4 SD), and Hardy-Weinberg test *P* < 1.0 × 10^−6^. The following criteria were used to exclude low-quality samples: call rate<95%, genetic sex being ‘male’, genetic ancestry not European, showing cryptic relatedness with others. It was imputed by minimac4 (version 1.0.0, https://genome.sph.umich.edu/wiki/Minimac). After imputation, we only retained SNPs with an MAF > 5%, high imputation quality (R^2^ ≥ 0.8), and available in the GWAS summary statistics from the Breast Cancer Association Consortium (BCAC) [4] and 1000 Genome Project 3, Phase 5 [31].

### Proteomics data processing

A total of 6388 proteins were detected in normal breast tissue samples from 120 women with a high detection false discovery rate (FDR < 0.01). A total of 5820 proteins were detected in at least 80% of the participants. Among them, the corresponding genes of 5616 proteins were located on auto chromosomes and were kept for downstream analyses. Probabilistic estimation of expression residual (PEER) factors was estimated via *peer* package in R, which is a software package using statistical models to improve the sensitivity and interpretability of genetic associations [34]. Protein expression levels were log2-transformed and then normalized via quantile and rank-based inverse transformations. To remove potential batch effects and experimental confounders, normalized protein expression levels were then regressed on age, the top three principal components, and 15 PEER factors [34]. We calculated and adjusted for PEER factors because they enhance the accuracy of how genetic associations are interpreted in large-scale expression data. The residuals were then used for downstream model building.

### Building protein prediction models

For each of the 5616 proteins, SNPs situated within a 500 Kilobases (Kb) range, both downstream and upstream of each gene encoding the corresponding protein, were evaluated as potential predictors. The elastic net models (α = 0.5) were constructed to predict the expression level of each protein, implemented via the R *glmnet* package [35–37]. Elastic net is a regularization and variable selection method, and has the advantage when the number of predictors is bigger than the number of observations [38]. After variable selection, internal model validation was performed using five-fold cross-validation to correct for potential overfitting and estimate the model’s prediction performance. The correlation (R) between the observed and the predicted expression was calculated to evaluate prediction performance, and corresponding p-values were determined.

### Breast cancer GWAS summary statistics

Breast cancer GWAS summary statistics came from the Breast Cancer Association Consortium (BCAC), which includes 133,384 breast cancer cases and 113,789 controls of European descent [4]. In addition to all types of breast cancer, we conducted stratified analyses based on five intrinsic breast cancer subtypes: luminal A, luminal B, luminal B/HER2-negative, HER2-enriched, triple-negative breast cancer (TNBC), ER-positive, and ER-negative [6].

### Association analyses of genetically predicted protein levels with breast cancer risk

Only proteins with prediction models showing a cross-validation performance of R > 0.1 and *P* < 0.05 were included in downstream association analyses with breast cancer risk, which is based on the standards from a previously published study [39]. Each prediction model was applied to the breast cancer GWAS summary statistics of BCAC [4] using S-PrediXcan [29]. The methodology’s details are described in another publication [3]. The formula for this method is like the following formula:

$${Z}_{p} \, \approx \, {\sum}_{s \in {{Model}}_{p}}\left({W}_{{sp}} \frac{\widehat{{\sigma }_{s}}}{\widehat{{\sigma }_{p}}} \frac{\widehat{{\beta }_{s}}}{\widehat{{{se}}(\beta _{s})}}\right)$$

A Z-score was calculated to estimate the association between the predicted protein expression and breast cancer risk. In this context, *w*_*sp*_ represents the weight of SNP *s* used in predicting the expression of protein *p*. The term $\hat{{\beta }_{s}}$ and $\hat{{se}}({\beta }_{s})$ refer to the effect size of association and the standard error for variant *s* in breast cancer GWAS summary statistics, respectively. Additionally, $\hat{{\sigma }_{s}}$ and $\hat{{\sigma }_{p}}$ denote the estimated variances of variant *s* and the predicted expression of protein *p*, respectively. We only assessed the correlations between variants included in the prediction models for this study. For each specific subtype of breast cancer, a Benjamini-Hochberg FDR of < 0.1 was considered indicative of statistical significance.

### Conditional analyses of genetically predicted proteins by adjusting GWAS-identified breast cancer risk variants

To assess whether the significant proteins were influenced by their nearby (±1 Megabase [Mb]) GWAS-identified risk variants, we performed a genome-wide complex trait analysis, conditional and joint analysis [40]. GCTA-COJO was employed to conduct the conditional analysis using summary-level statistics from BCAC and estimated linkage disequilibrium from individual-level genotype data of European ancestry from the 1000 Genome project [40]. CGTA-COJO can estimate the $\hat{{\beta }_{s}}$ and $\hat{{se}}({\beta }_{s})$ with breast cancer risk after adjusting for the GWAS-identified variants [41]. After that, S-PrediXcan was used once more to estimate the predicted protein expression levels in association with breast cancer risk, taking into account the GWAS-identified variants for breast cancer [41]. The independent association between genetically determined breast-tissue proteins and breast cancer risk was defined as a *p* value reaching *P* < 1 × 10^–4^ after conditional analyses.

### Correlation analysis between protein expression and gene expression in Komen samples

Gene expression data of cancer-free breast tissue from the 120 EA individuals were generated via RNA-Seq in another project. Similar methods were described in detail in a previous publication [3]. The RNA-seq data were QCed and normalized by applying log2-transformation, removing genes missing in more than 80% of samples, and quantile normalization. Similarly, for protein expression data, we used log2-transformation, removed proteins missing in more than 80% of samples, and applied quantile normalization. We then performed a Spearman correlation analysis to examine the correlation between protein expression and gene expression, utilizing the *cor.test* function available in the *stats* R package.

## Results

### Breast tissue protein expression prediction model building

**Fig. 1:**
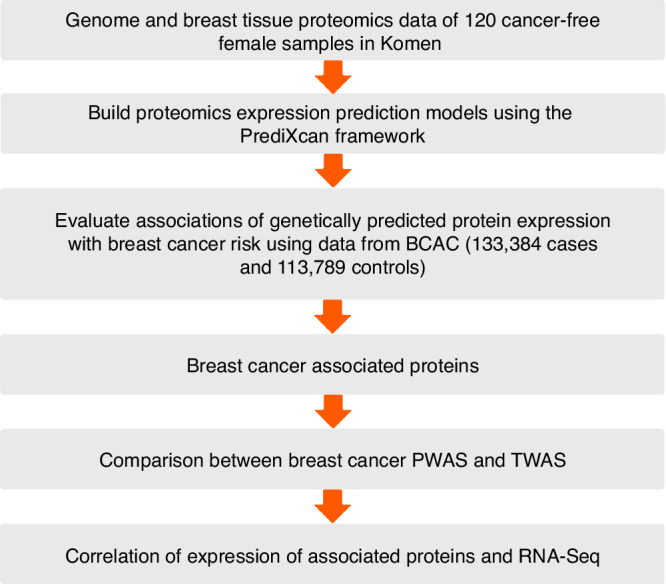
Study design flow chart. Genome and breast tissue proteomics data were obtained from 120 cancer-free female samples in the KTB database. Proteomics expression prediction models were then built using the PrediXcan framework. Associations between genetically predicted protein expression and breast cancer risk were evaluated using GWAS summary statistics from BCAC, including 133,384 cases and 113,789 controls. Proteins associated with breast cancer risk were identified. Subsequently, a comparison between breast cancer PWAS and TWAS was performed, followed by a correlation analysis between protein expression and corresponding RNA-Seq data.

The overall study design is presented in Fig. 1. Among the 5820 proteins passing quality control, prediction models were successfully built for 2060 (35.4%) proteins with R > 0.1 and *P* < 0.05. The selection of the number of PEER factors was based on analyzing up to 25 PEER factors in increments of five, as shown in Supplementary Fig. 1. The number of models built was deemed sufficient when the number of PEER factors exceeded five and reached the maximum at 15; therefore, in this study, we chose 15 PEER factors (Supplementary Fig. 1). In the downstream association analyses, we will focus on the 2060 proteins with prediction R > 0.1 and *P* < 0.05.

**Fig. 2:**
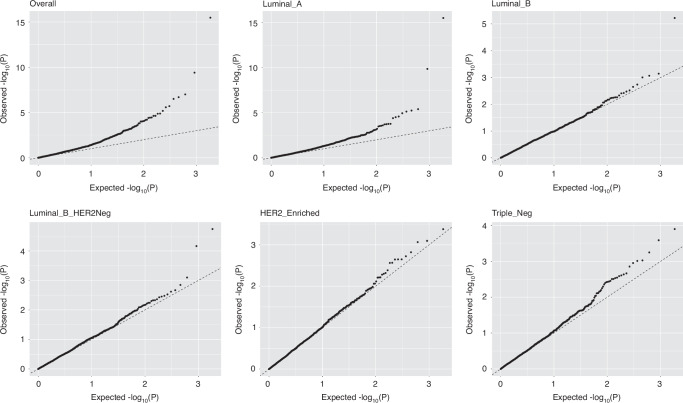
Quantile-quantile plots for the PWAS. Q-Q plots of -log10-transformed p-values versus expected p-values under the null hypothesis for overall breast cancer and its subtypes.

### Associations of genetically predicted protein expression in breast tissue with overall breast cancer risk

Figure 2 displays the Q-Q plot comparing the -log10-transformed *p* values from our association study of breast cancer risk against the expected -log10-transformed p-values under the null hypothesis. In the plot, the deviation of association signals from the null hypothesis line suggests that a few proteins are significantly associated with overall breast cancer or its subtypes. This aligns with our results presented in Table 1, where several proteins showed a significant association with breast cancer risk after FDR correction.

**Table 1:** Seven unique proteins are associated with breast cancer risk with FDR-corrected P < 0.1.

Cancer	Region	Protein	Lead GWAS Variant(Hg37)	Distance (kb)^b^	Prediction Model		Association with Breast Cancer			
					R	SNPs	Z	P	FDR	P_adjusted_
Overall	3q21.3	COPG1	–	–	0.21	17	3.33	8.54 × 10^–4^	0.06	–
	9p13.3	DCTN3	–	–	0.27	40	–3.29	1.01 × 10^–3^	0.06	–
	11p15.5	LSP1^c^	11:1896957:G:C^b^	16.54	0.38	40	4.13	3.63 × 10^–5^	0.01	0.62
	11q23.3	DDX6	–	–	0.20	39	–3.59	3.25 × 10^–4^	0.03	–
	16p13.3	DNAJA3	16:4027749:A:G^b^	448.06	0.18	7	–3.91	9.15 × 10^–5^	0.01	1.94 × 10^–4^
Luminal A	3p21.31	SMARCC1	3:46889187:G:A^b^	737.58	0.19	48	3.33	8.68 × 10^–4^	0.09	0.23
	11p15.5	LSP1^c^	11:1896957:G:C^b^	16.54	0.38	40	3.59	3.31 × 10^–4^	0.05	0.56
	12q13	NCKAP1L^a^	–	–	0.23	9	3.75	1.74 × 10^–4^	0.05	–
Luminal B	11p15.5	LSP1^c^	11:1896957:G:C^b^	16.54	0.38	40	4.53	6.01 × 10^–6^	1.79 × 10^–3^	6.43 × 10^–3^
ER-Positive	11p15.5	LSP1^c^	11:1896957:G:C^b^	16.54	0.38	40	4.19	2.79 × 10^–^^5^	8.30 × 10^–3^	0.29

^a^Have been reported in an unpublished TWAS study by Ping et al.

^b^Identified by Jia et al. [3].

^c^Have been reported in two TWAS studies by Gao et al. and Li et al. [13, 18].

By analyzing 2060 reliably predicted protein models, we identified five proteins associated with overall breast cancer risk at *P* < 1.01 × 10^–3^, an FDR-corrected significance level <0.1 (Table 1). Among these five proteins, the corresponding genes for three proteins, including *COPG1*, *DCTN3*, and *DDX6*, were located at least 1Mb away from the GWAS-identified breast cancer risk variants. Increased protein expression levels were associated with a decreased risk of breast cancer for DCTN3 and DDX6, with *p* values of 1.01 × 10^–3^ and 3.25 × 10^–4^, respectively. For the protein COPG1, an increased level was associated with increased breast cancer risk, with a *p* value of 8.54 × 10^–4^.

For the remaining two proteins, LSP1 and DNAJA3, the corresponding genes were in previously GWAS-identified breast cancer risk loci. A positive association between the genetically predicted levels of the LSP1 protein and breast cancer risk was observed (*p* value of 3.63 × 10^–5^), while DNAJA3 showed an inverse association with breast cancer risk, with a *p* value of 9.15 × 10^–5^. After adjusting for GWAS-identified risk variants (16:4027749:A:G, reported by Jia et al. [3]), the associations for DNAJA3 were still significant (adjusted *p* value of 1.94 × 10^–4^), however, the association for LSP1 became insignificant (adjusted *p* value of 0.62).

### Associations of genetically predicted protein expression with breast cancer subtypes

We conducted stratification analyses to investigate the genetically predicted protein levels in association with breast cancer subtypes (Table 1). The associations of the seven proteins with subtypes of breast cancer are shown in Tables 2, 3. We observed significant associations for ER-positive (LSP1), lumina A (SMARCC1, LSP1, NCKAP1L), and lumina B subtypes (LSP1).

**Table 2:** Association between breast cancer risk in intrinsic subtypes and seven significant proteins mentioned in Table 1.

Region	Protein	Overall		Luminal A		Luminal B		Luminal B/HER2-negative		HER2-enriched		Triple-negative	
		Z	P	Z	P	Z	P	Z	P	Z	P	Z	P
3p21.31	SMARCC1	3.03	2.42 × 10^–3^	3.33	8.68 × 10^−4^	–0.05	9.62 × 10^–1^	1.06	2.88 × 10^–1^	1.16	2.48 × 10^–1^	1.05	2.92 × 10^–1^
3q21.3	COPG1	3.33	8.54 × 10^–4^	2.47	1.35 × 10^−2^	2.71	6.72 × 10^–3^	0.29	7.69 × 10^–1^	0.67	5.03 × 10^–1^	2.35	1.90 × 10^–2^
9p13.3	DCTN3	–3.29	1.01 × 10^–3^	–2.69	7.22 × 10^−3^	–0.55	5.82 × 10^–1^	–1.85	6.48 × 10^–2^	–1.08	2.79 × 10^–1^	–1.85	6.43 × 10^–2^
11p15.5	LSP1^b^	4.13	3.63 × 10^–5^	3.59	3.31 × 10^−4^	4.53	6.01 × 10^–6^	0.65	5.18 × 10^–1^	1.60	1.10 × 10^–1^	0.33	7.43 × 10^–1^
11q23.3	DDX6	–3.59	3.25 × 10^–4^	–2.76	5.83 × 10^−3^	0.02	9.80 × 10^–1^	–2.41	1.62 × 10^–2^	–0.46	6.47 × 10^–1^	–1.37	1.71 × 10^–1^
12q13	NCKAP1L^a^	2.72	6.48 × 10^–3^	3.75	1.74 × 10^−4^	–0.03	9.80 × 10^–1^	1.71	8.68 × 10^–2^	1.99	4.66 × 10^–2^	–0.53	5.96 × 10^–1^
16p13.3	DNAJA3	–3.91	9.15 × 10^–5^	–3.16	1.60 × 10^−3^	–2.51	1.21 × 10^–2^	–1.64	1.00 × 10^–1^	–0.31	7.57 × 10^–1^	–1.72	8.47 × 10^–2^

^a^Have been reported in an unpublished TWAS study by Ping et al.

^b^Have been reported in two TWAS studies by Gao et al. and Li et al. [13, 18].

**Table 3:** Association between ER-positive and ER-negative breast cancer risk and seven significant proteins mentioned in Table 1.

Region	Protein	Overall		ER-Positive		ER-Negative	
		Z	P	Z	P	Z	P
3p21.31	SMARCC1	3.03	2.42 × 10^–3^	3.36	7.77 × 10^–4^	0.47	6.35 × 10^–1^
3q21.3	COPG1	3.33	8.54 × 10^–4^	2.75	5.92 × 10^–3^	2.44	1.46 × 10^–2^
9p13.3	DCTN3	–3.29	1.01 × 10^–3^	–2.88	3.99 × 10^–3^	–1.93	5.34 × 10^–2^
11p15.5	LSP1^b^	4.13	3.63 × 10^–5^	4.19	2.79 × 10^–5^	1.49	1.37 × 10^–1^
11q23.3	DDX6	–3.59	3.25 × 10^–4^	–2.41	1.58 × 10^–2^	–1.48	1.39 × 10^–1^
12q13	NCKAP1L^a^	2.72	6.48 × 10^–3^	2.69	7.16 × 10^–3^	0.00	9.99 × 10^–1^
16p13.3	DNAJA3	–3.91	9.15 × 10^–5^	–3.20	1.39 × 10^–3^	–1.69	9.07 × 10^–2^

^a^Have been reported in an unpublished TWAS study by Ping et al.

^b^Have been reported in two TWAS studies by Gao et al. and Li et al. [13, 18].

For ER-positive, we also observed that protein LSP1 was significantly positively associated with breast cancer risk, with a *p* value of 2.79 × 10^–5^. It was located within 1Mb of GWAS-identified variants (11:1896957:G:C, reported by Jia et al. [3]). After adjusting for the variants, the significance of LSP1 became weaker (adjusted *p* value of 0.29).

For lumina A, SMARCC1, LSP1, and NCKAP1L were positively associated (*p* values of 8.68 × 10^–4^, 3.31 × 10^–4^, and 1.74 × 10^–4^, respectively). The genes *SMARCC1* and *LSP1* were located within 1Mb of GWAS-identified risk variants, but the gene *NCKAP1L* was not close to any known risk variants. The significance of proteins SMARCC1 and LSP1 became weak after adjusting for nearby GWAS-risk variants (adjusted *p* values of 0.23 and 0.56, respectively).

For luminal B subtype, we observed a significantly positive association for LSP1 with a *p* value of 6.01 × 10^–6^. After adjusting for its nearby risk variant (11:1896957:G:C, reported by Jia et al. [3]), the significance weakened, resulting in a *p* value of 6.43 × 10^–3^.

### Comparing the results of PWAS and TWAS

For the promising proteins associated with breast cancer risk shown in Table 1, we compared the associations between predicted protein expression with breast cancer risk, and the associations of predicted gene expression levels with breast cancer risk (Table 4). Among seven proteins, one corresponding gene was associated with breast cancer risk at a *p* value of <0.05, and two with a consistent direction. NCKAP1L demonstrated a positive association with breast cancer risk in both PWAS and TWAS, with respective *p* values of 1.74 × 10^–4^ and 6.77 × 10^–4^. DDX6 and DNAJA3 both exhibited a negative association with breast cancer risk in this study. However, their p-values from TWAS were not less than 0.05.

**Table 4:** Comparison between breast cancer PWAS and TWAS for identified proteins shown in Table 1.

Region	Protein	PWAS			TWAS	
		Z	P	FDR	Z	P
3p21.31	SMARCC1	3.33	8.68 × 10^–4^	0.09	–	–
3q21.3	COPG1	3.33	8.54 × 10^–4^	0.06	–	–
9p13.3	DCTN3	–3.29	1.01 × 10^–3^	0.06	–	–
11p15.5	LSP1^2^	4.13	3.63 × 10^–5^	0.01	–	–
11q23.3	DDX6^a^	–3.59	3.25 × 10^–4^	0.03	–0.73	0.47
12q13	NCKAP1L^a^	3.75	1.74 × 10^–4^	0.05	3.40	6.77 × 10^–4^
16p13.3	DNAJA3^a^	–3.91	9.15 × 10^–5^	0.01	–0.40	0.69

^a^Have been reported in an unpublished TWAS study by Ping et al.

### Correlations between protein expression levels and gene expression levels in normal breast tissue

For the promising proteins that are associated with breast cancer risk in Table 1, we investigated the correlations between the protein levels and the gene expression levels in normal breast tissue (Table 5). Among the seven proteins, five were positively correlated (SMARCC1, COPG1, LSP1, NCKAP1L, DNAJA3) and two (DCTN3, DDX6) were negatively correlated. Among those positively correlated, the correlation rho ranged from 0.10 to 0.61, with a median rho of 0.31.

**Table 5:** Spearman correlations between protein and gene expression.

Region	Protein		Spearman correlation		
		Rho	95% CI	P	FDR
3p21.31	SMARCC1	0.61	(0.59, 0.62)	3.81 × 10^–6^	2.22 × 10^−5^
3q21.3	COPG1	0.31	(0.29, 0.33)	6.30 × 10^–4^	2.18 × 10^−3^
9p13.3	DCTN3	–0.21	(–0.24, –0.19)	0.02	4.36 × 10^−2^
11p15.5	LSP1^b^	0.25	(0.23, 0.28)	5.28 × 10^–3^	1.41 × 10^−2^
11q23.3	DDX6	–0.07	(–0.10, –0.05)	0.44	0.55
12q13	NCKAP1L^a^	0.35	(0.32, 0.37)	3.49 × 10^–3^	9.85 × 10^−3^
16p13.3	DNAJA3	0.10	(0.07, 0.12)	0.31	0.42

^a^Have been reported in an unpublished TWAS study by Ping et al.

^b^Have been reported in two TWAS studies by Gao et al. and Li et al. [13, 18].

## Discussion

In this first breast-tissue-based PWAS of breast cancer risk, we found that five proteins (COPG1, DCTN3, LSP1, DDX6, and DNAJA3) were significantly associated with overall breast cancer risk. COPG1 and DCTN3 were not reported in previous studies, including TWAS and blood-based PWAS. COPG1 was not reported in TWAS or PWAS for breast cancer, but reducing the expression of the *COPG1* gene lessened the accumulation of full-length nuclear c-MET, a receptor tyrosine kinase commonly overexpressed in various malignant cancers, including breast cancer [42].

*DCTN3* is responsible for encoding the smallest (p22/24) subunit of dynactin, which is a cytoplasmic motor protein complex that plays a crucial role in cellular processes, including chromosome movement and nuclear positioning [43, 44]. We found that genetically determined DCTN3 protein levels were inversely associated with the risk of breast cancer. Previous research indicates that DCTN3 overexpression may play a role in breast cancer progression [43]. These findings indicate that our results are consistent with prior studies. Additionally, our study suggests that the gene DCTN3 is negatively correlated with its protein expression. Complex regulatory processes take place at each stage of transcription and translation, and many factors could affect mRNA stability, mRNA-to-protein translation, post-translation regulations, and protein stability [45]. As a result, overall, protein levels and mRNA levels are only moderately correlated. The negative correlation between a protein and its corresponding gene expression is common and has been observed in previous studies [46, 47]. In the GTEx project, data from 10,349 genes and their corresponding proteins, generated from the same specimens, were analyzed [46]. Using an FDR threshold of less than 0.05, the study found that 6228 genes showed a positive correlation between protein and RNA levels across tissues [46]. Conversely, 60 genes exhibited a negative correlation, while the remaining 4061 genes did not show a significant correlation [46]. The median Spearman correlation between the mRNA levels and the corresponding protein levels was only 0.46 [46]. Another study profiled 92 protein levels measured in 2,014 European ancestry whole blood samples and identified seven proteins that showed negative correlations with their respective gene expression across multiple tissues [47]. The researchers suggested that these observed negative correlations need further investigation to unravel the underlying post-transcriptional or pathway-associated regulatory processes [47]. *DCTN3* undergoes post-translational modifications, including ubiquitination at Lys50 and Lys156 [48]. These modifications may contribute to the observed negative correlation between its gene and protein expression. DDX6 is located more than 1Mb away from nearby risk variants, and DNAJA3 is still significant after conditioning on nearby risk variants. *DDX6* is part of the DEAD box proteins, which are putative RNA helicases, and is implicated in (11;14) (q23;q32) translocation in B-cell lymphoma [49, 50]. It is commonly amplified in breast cancer nodal metastases [49]. *DNAJA3* functions as a mitochondrial co-chaperone with a conserved DnaJ protein domain that is essential for interacting with the ATPase activation in heat shock protein 70 [51]. In physiological processes, *DNAJA3* plays a critical role in protein folding, assembly, translocation, and degradation during cell growth and development [51–54]. *DNAJA3* was significantly coexpressed with *PALB2* in breast cancer [55].

Analyses stratified by breast cancer subtypes found that SMARCC1, LSP1, and NCKAP1L are associated with luminal A and LSP1 with luminal B and ER-positive subtype. SMARCC1 was not reported in previous TWAS or PWAS studies for breast cancer. NCKAP1L was located 1Mb away from the risk lock, and LSP1 retained statistical significance after adjusting for nearby risk variants. In a previous TWAS study, *LSP1* was not identified as associated with breast cancer risk in breast tissue samples, but it was identified as negatively associated with breast cancer risk in cultured fibroblast cells [13]. In a multi-tissue TWAS study, *LSP1* was identified as associated with luminal A and luminal B subtypes [18]. Moreover, in a gene-based aggregation study, it was reported to be significantly associated with breast cancer risk and was implicated in the etiology of other cancer types [56]. This confirmed the consistent association between LSP1 protein and breast cancer risk in the current study. The *SMARCC1* gene plays the role of a core subunit in the SWI/SNF chromatin remodeling/tumor suppressor complex, and the function of *SMARCC1* is influenced by *CARM1*, a protein arginine methyltransferase [57]. *CARM1* specifically methylates *SMARCC1*, which is a key modification that aids in the targeting of *SMARCC1* to genes in the c-Myc pathway [57]. This targeting is significant as it enhances breast cancer progression and metastasis [57]. Therefore, *SMARCC1* is pivotal as its modification by *CARM1* directly impacts gene regulation and cancer progression. *NCKAP1L* is an important part of the actin cytoskeleton machinery, and is a hematopoietic lineage-restricted member of the Nap1l subunit of the WASP-family verprolin-homologous protein complex [58]. The messenger RNAs of *NCKAP1L* play a prognostic role in the tumor microenvironment of luminal breast cancer; higher NCKAP1L is associated with better prognostic ability [59]. In an unpublished TWAS by Jie et al., *NCKAP1L* was found to be positively associated with breast cancer risk, which aligns with our findings.

Correlation analysis revealed that SMARCC1, COPG1, LSP1, and NCKAP1L were positively associated with their corresponding genes. Observing strong correlations is expected. However, a low correlation could be attributed to post-translational modifications, highlighting the necessity of PWAS. Besides, only NCKAP1L was reported in an unpublished TWAS as being associated with breast cancer risk. This highlights the advantage of PWAS over TWAS, as it helps identify associations not reported in TWAS, showing its capabilities beyond what TWAS can achieve.

One limitation of our study is the exclusive inclusion of cancer-free breast tissue samples from individuals of European descent. Conducting a multi-ancestry breast tissue PWAS would be beneficial for identifying candidate protein biomarkers for breast cancer. Unfortunately, there are currently no publicly available cancer-free breast tissue samples from other ethnicities that we could incorporate to explore the applicability of our findings to women of non-European ancestries. Additionally, the majority of participants in the KTB are of European ancestry. Due to budget constraints and the limited availability of normal breast tissue samples, only women of European ancestry were included in the present study. The generalizability of our findings in other ethnic populations needs to be investigated in future studies. Another limitation is the limited sample size and lack of external validation. The high costs of obtaining them and conducting genotyping and protein expression profiling pose some challenges. In our first TWAS, we used the gene expression data in normal tissue samples from 60 women to build gene expression models [16]. We identified 48 genes [16], and 7 of the genes were replicated in later studies with larger sample sizes [13]. In the future, we will try to generate more data in normal breast tissue samples and validate the prediction models in the current study. Additionally, we used FDR p-values of < 0.1, which is less stringent than approaches like Bonferroni-corrected p-values. Given our relatively small sample size of 120 samples, a less stringent method is preferred. This study is exploratory, being the first PWAS using tissue data instead of blood data, so a less strict criterion is understandable. In the present study, an untargeted LC-MS/MS approach was employed to detect all peptide signals. Unlike targeted approaches, untargeted methods allow for the comparison of the same protein or peptide across different samples. However, a notable disadvantage of untargeted quantification is its inability to compare protein abundance across different proteins. This limitation stems from the fact that the untargeted approach generates relative quantification based on signal peak intensity, and different proteins or peptides can produce varying signal patterns within the instrument. Consequently, comparing protein abundance among different proteins using relative quantification does not rely on a consistent baseline. In contrast, targeted approaches enable comparisons between different proteins by converting signal intensity to actual protein quantities. We preferred the untargeted approach in our study because it allowed us to build a prediction model for each protein across all samples, facilitating the comparison of the same protein among different samples. Quantifying protein levels using mass spectrometry presents several challenges, primarily due to insufficient signal clarity. To address this issue, we included all available samples within our funding constraints to improve the signal-to-noise ratio. Also, we employed high-resolution mass spectrometry instruments and utilized liquid chromatography columns to fractionate samples, thereby enhancing detection accuracy.

## Conclusion

We conducted the first breast-tissue-based PWAS and identified seven proteins associated with breast cancer risk, including five proteins not previously reported. These findings help improve our understanding of the underlying genetic mechanism of breast cancer development.

## Supplementary information

Supplementary Figure Legend

Supplementary Figure 1

## Data Availability

The data that support this study are available from the corresponding author upon reasonable request.

## Code availability

Publicly available software and packages were used throughout this study according to each developer’s instructions. Custom scripts generated for the study will be available upon request.
