# Supplementary material for: A proteome-wide association study identifies putative causal proteins for breast cancer risk
Source: Br J Cancer. 2024 Oct 28;131(11):1796–804. doi: 10.1038/s41416-024-02879-1 (PMC11589835; doi:10.1038/s41416-024-02879-1)
Supplement: Supplementary file 1 — Supplementary Figure Legend [file 41416_2024_2879_MOESM1_ESM.docx]

**Supplementary Figure Legend**

**Supplementary Figure 1**. Number of protein prediction models built versus number of PEER factors used in models.
