# Supplementary figures and images for: A proteome-wide association study identifies putative causal proteins for breast cancer risk
Source: Br J Cancer. 2024 Oct 28;131(11):1796–804. doi: 10.1038/s41416-024-02879-1 (PMC11589835; doi:10.1038/s41416-024-02879-1)

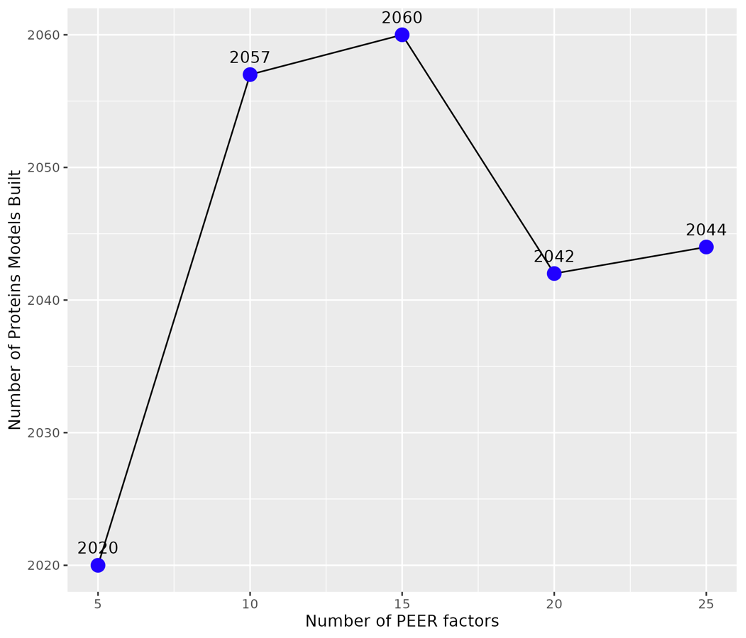

Supplement: Supplementary file 2 — Supplementary Figure 1 [file 41416_2024_2879_MOESM2_ESM.png]
